# Periodicity
**Health and Disease as Influenced by the Daily, Seasonal, and other Cyclical Changes in the Human System.* By Edward Smith, M.D., LL.B., F.R.S. 8vo. pp. 409. London. 1861.


**Published:** 1862-01

**Authors:** 


### ART. III.—PERIODICITY.*

WE learn from the early English metrical romances relating to King Arthur, that Sir Gawain, one of the most redoubted of the valiant knights of the Round Table, possessed (thanks to a saintly benediction) a very peculiar privilege. A man of mighty thews and sinews, his strength at all times surpassed that of common men, but it was subject to considerable oscillations, depending upon the diurnal progress of the sun. Thus from *overtime,* that is to say, nine o'clock in the morning, until noon, Sir Gawain's muscular powers were doubled; from thence until three o'clock in the afternoon they relapsed into their ordinary state; from three

---

* *Health and Disease as Influenced by the Daily, Seasonal, and other Cyclical Changes in the Human System.* By Edward Smith, M.D., LL.B., F.R.S. 8vo. pp. 409. London. 1861.

until evensong they were again doubled; after which this preternatural accession of strength once more subsided until day-break.

Remarkable as this phenomenon might at the first aspect appear, it holds but a very modest rank among the many marvels which distinguished the epoch of knight-errantry. Rarely did the supernatural in that mythical period condescend to ally itself so closely with the natural. Swords tempered in no earthly fires and of irresistible edge (witness the Escalibore of King Arthur, and the Aroundight of Sir Lancelot), mystic rings, which shielded the possessor from enchantment, and from danger by fire and by water, impenetrable armour, and magical cantrips of sundry descriptions, were the aids by which heroic distinction was chiefly acquired among Sir Gawain's compeers; but his own marvellous powers were brought about by the simple exaggeration of a natural phenomenon.

That the harmonious and periodical changes observed in the position of the sun and other heavenly bodies to the earth, were intimately reflected in man as well as in the varied phenomena occurring on the surface of the globe, was a belief entertained from the remotest period of which we have any definite account. It has been handed down to us, indeed, as a necessary assumption from matters of ordinary observation. Night and morning, the progress of the months, the succession of seasons, and the revolutions of the moon, on the one hand ; and on the other, the diurnal occurrence in man of sleeping and waking, of fatigue and renovation, of appetites and wants, the greater liability to certain diseases at certain seasons, and the menstrual flux in woman, formed a wide foundation on which to build the assumption that the periodical changes going on in the human frame were closely linked to the periodical changes in the position of the heavenly bodies to the earth, and among themselves. Broadly stated, this assumption is simply a truism ; and if we permit ourselves to laugh at the extravagancies into which it often led the philosophers and physicians of the early and middle ages, in their attempts to trace the connexion between every variation in man's state and condition with the changes observed in the position of the sun and the moon, the stars and the planets, let us also take shame to ourselves that modern science has hitherto done so little to follow out the consequences of the great truth which lies under the assumption.

Truly it was a pleasant conceit that the whole history of man from the cradle to the grave was figured within the narrow bounds of a single day. In the morning he arises flushed with renovated vigour—at mid-day his powers culminate—in the evening his matured forces wane—and in the night he gladly seeks repose for his wearied frame in sleep. The morning thus reflects the periods of youth and joy, of sanguine vigour (the blood predominating), and of spring; mid-day represents virile age, the epoch of ardent passions (the bile predominating), and summer; the evening, maturity and sadness (the atrabilious period), autumn; and night, old age and the first infancy, (the time of cold and moist humours, *pituita,*) winter. Moreover, from infancy to old age, the liability of different parts of the body to disease differs, and increases in a definite order as years advance, proceeding from above downwards. Thus, during infancy the head is most subject to disease, in adolescence the neck and chest, in manhood the stomach, liver, and associated viscera, and in old age, the urinary organs, and hœmorrhoidal veins. As a consequence, the invasion and exacerbations of the diseases of different parts of the body are most apt to occur during those periods of the day which are the counterparts of the epochs of life to which the maladies are most peculiar.

Fanciful as these conceits may be, there is nevertheless beneath them a sound kernel, which has not yet been fully extracted. Observation has abundantly shown that the inception, exacerbations, and terminations of many diseases do not occur indifferently at any period of the day.

Physicians in the past century, as well as those of a more remote date, gave somewhat more attention to the periodical phenomena of disease than we are accustomed to do at the present day; and it may not be amiss, in illustration of a much neglected subject, to quote, in part, Virey's admirable (but not generally accessible) summary,* at the beginning of the present century, of the results of observation on the influence of the diurnal revolution of the earth upon maladies.

"Let us transport ourselves," he writes, "to the sad asylums of human infirmities. If their vaults repeat unceasingly the groans of the sick, yet the pangs of the sufferers are not constantly the same at all hours, and death walks among their ranks with unequal steps day and night.

*Morning* [3 a.m. to 9 a.m.]. It is known that the majority of diseases undergo a remission in the morning, according to the axiom, *levato sole, levatur morbus* (Bayer, *Adag. medicin. centur.* Francfort, 1718, in 8vo), from the effect of the reparation produced by sleep. This remission is such in mucous fevers, intermittents especially, tertians and double tertians, that individuals in agony during the entire night will rise with the sun in the morning and recover sufficient vigour to go about (Rammazzini, *Constit. epid. mutin.*, art. x. Operum, p. 126, edit. Genev., 1717, in 4to). Moreover, the insensible perspi-

* See *Dictionnaire des Sciences Médicales*, vol. xxvi. art. *Jour.*

ration, then more abundant, generally solaces many. Thus the oppression of the dropsical and œdema of the legs become less ; and hectic fever ceases at this epoch alone of the nyctemeron [the twenty-four hours of night and day]. The functions of the nervous system being re-invigorated by the night's rest, a remission in nearly all spasmodic diseases is induced. The majority of inflammatory disorders of the mucous membranes, such as catarrhs and croup, also abate. Finally, there are few affections with an evening exacerbation which have not an intermission during the morning. The progress also of the majority of asthenic disorders is then moderated, on account of the greater vigour of the organization at this epoch.

"But this matutinal vigour becomes the source of invasion, and of the paroxysms of many sthenic affections. For example, angiotenic (synochal) fever has an early morning invasion (Pinel, *Nosog.*, tom. i., p. 29, edit. iv.) ; the eruptions of the exanthemata ordinarily appear at this epoch, particularly among children ; quinsey (which has an evening exacerbation) attacks at this time ; the accession of bilious or gastric fever, remittent or intermittent, whether following the quotidian, tertian, or double tertian type, or being erratic, always occurs before mid-day. In the so-called putrid fevers, the adynamia often manifests itself in the morning. The colliquative sweats of the phthisical, and hysterical swellings, increase also at this period (Sydenham, *Dissert. epist.*, p. 41 ; Raulin, *Malad. vapor.* p. 221), and worms torment then, doubtless on account of the intestines being empty ; from this cause also happens pyrosis or *soda.* In the morning ophthalmic inflammations appear more acute, and the hæmoptysis of young men is commonly manifested. In general inflammatory affections of the throat, vernal fevers, quotidian or tertian, simple synochal fevers, and many other plethoric maladies of spring and youth, tend evidently to show themselves and become aggravated in the first hours of the day. These are moreover, for the most part, affections of the parts above the diaphragm.

In malignant or ataxic fevers, particularly typhus, there are two exacerbations in the day ; but that of the morning is more violent than that of the evening, as Hufeland remarked. Wounds also, gangrenous ulcers, carcinomatous affections, and highly inflamed phlegmons, undergo an augmentation of heat, pain, and tension at sunrise.

*Mid-day* [9 a.m. to 3 p.m.]. In proportion as the sun approaches the zenith, bilious affections, and strong nervous emotions in mania and hydrophobia, grow worse ; Sauvages has termed one of these affections, which occurs only during the day and disappears at night, *solar mania* (*see* also *Eph. nat. cur.*, dec. iii., obs. 32, an. iii.). This author (*Nosolog.*, art. *dæmonomania hysterica*) cites moreover a female who began to act extravagantly precisely at one hour after noon, although they endeavoured to deceive her as to time. Phrenetics become excited in an especial manner towards two or three of the afternoon, and are afflicted with remarkable trembling and exacerbations (Pinel, *Nosog.*, tom. iv., p. 306, ed. iv.). Musgrave records an acute arthritic cephalic affection which recurred every day at noon, and Sauvages notes an analogous example. Ordinarily the eruptive fever of discrete small-pox appears about the same hour. Coma *vigil*, typhomania (*dem el muya* of the Egyptians), calenture, the violent delirium of malignant remittent fevers of hot countries, yellow fever, tetanus and trismus, erysipelas, *coups de soleil*, manifest themselves much more frequently in the heat of the day than at other times—sometimes also they show themselves before noon (Romans, *Natural History of Florida*, p. 247). Cholera morbus, the spasmodic vomitings in the neuroses of digestion (Pinel, *Nosogr.*, iii., p. 202), undergo their exacerbations towards noon, as well as colics, volvulus, and many gastric tertian fevers, &c. Lastly hepatitis, gastritis, the bilious diarrhœas of spring, among men of an irritable complexion, are more particularly augmented towards the middle of the day. Active hæmorrhages also come in the meridian heat.

"*Evening.* Fernel (*Liber de abditis morborum causis*) had remarked that all quartan fevers recur solely in the evening, as the sanguine quotidians at sunrise, and the tertians towards noon. But it is towards evening that in an especial manner the paroxysms of a host of maladies multiply. All those of a catarrhal character; the heavy pains of phlegmons, of inflammations, of the organs of animal life or of relation, increase astonishly in the evening, without doubt from the weakening of the exterior life; also cephalalgias or migraines augment much then, and comatose affections, and apoplexies not *foudroyant* rarely occur but in the evening or the night; paralytic affections, lethargy, tremors, syncope, the unaccustomed emotions of hypochondriasis and of hysteria, low nervous fevers, the oppression of dropsy, the swelling of œdemas, articular pains, facial and femoro-popliteal neuralgia (sciatica), necessarily become aggravated by this weakening of the sensitive life. An intermittent febrile hemiplegia has been seen to commence at four in the evening and cease at six in the morning (Torti, *De febribus*, c. iv., p. 227), cured by quinquina; a periodical cough at seven in the evening stopped by means of opium (Darwin, *Zoonom.*, tom. ii., sect. 36). It is in the evening more especially that suppurative fever in the wounded is set up; and jactitation and inquietude are peculiar to a multitude of nervous lesions at this epoch. When hæmorrhages (epistaxis, hæmorrhoids, &c.) supervene at this time, they are nearly always the result of a spasm, which without doubt also causes that insupportable anxiety suffered by the phthisical, when they have a vomica. Cutaneous maladies—the itch, herpetic, impetiginous, and other pustular, vesicular, and papular affections (*les dartres*), and chilblains are likewise more troublesome in the evening, and it would be easy to multiply examples of these vespertine exacerbations.

"We might think that the light of the day, the work of the senses, the reception of new chyle into the blood in consequence of the nutriment taken, the irritation even of remedies, would dispose the economy in the evening to this general movement of exacerbation; nevertheless, although we may sleep all day, and follow an exact diet, hectic fever recurs not the less at its accustomed hour. On the other hand, we observe affections of the throat and some other morning affections become dissipated in the evening.

"As a rule, affections of the organs beneath the diaphragm, among men of mature age, as, for example, of the urinary passages, hæmorrhoids, arthritis, melancholy, dysentery, engorgements of the viscera, the spleen, and of the mesentery, obscure chronic maladies in the hypogastrium, as in a cavern of ills, are particularly aggravated at this period of the day, as during the autumn (Hippocr. *Aphor.*, sect. iii., § 23, et lib. iv. *Epidemior. Quale est ad vesperam exacerbari, ita et in omni morbo*).

"*Night.* Already many observers (see *Recueil de Mem. couronnés par la Société méd. de Bruxelles*, 1806, 8vo, *Mem.* de Laprade, *sur l'influence de la nuit*, de Moricheau Beauchamp, 1808; *l'Essai* de Ch. J. E. Guillaumod, Paris, 1812, 4to, and many others) have directed attention to the effects of the night upon man healthy and ill; but they have not considered, as seems to me, either the nocturnal epochs, or the general revolution. They comprehend rightly that sthenic affections, increasing during the day, undergo a remission from the nocturnal cold, obscurity, and humidity; and that, on the contrary, maladies which are better during the day, as fevers, mucous phlegmasiæ, catarrhs, anginæ, croup, affections of the lymphatic system, dropsy, cachexy, asthenic and adynamic maladies, and paralytic affections in general, will become aggravated in the night. There are occasionally particular states of our organs at divers hours. M. Humboldt cites (*Exper. sur l'irritabilité des muscles et des fibres nerveuses*, t. ii., p. 185) a countess of Madrid, who lost her voice at sunset and recovered it at sunrise. This peculiar paralysis of the recurrent nerves of the eighth pair disappeared in the climate of Naples, and reappeared in that of Rome. Other nocturnal paralyses, as well as deliriums and a vertigo, have been recorded (*Eph. nat cur.*, dec. ii., art. iv., p. 63) at the same epoch. This gives colour to the case related by Aristotle (*De mirabil. auscult.*) of an innkeeper at Tarentum, who was very rational during the day, but who always became mad at nightfall. Hemeralopic individuals undergo also then very sensibly that singular collapse which prevents them seeing, whilst nyctalops, on the contrary, see better by a feeble light. In like manner catalepsy has been noted as to occur during the day, but to pass away at night (Sauvag. *Nosol. catochus diurnus*), and certain cephalalgias to commence, others to cease at these epochs. . . ."

Without insisting upon the absolute correctness of several of the observations summed up by Virey, it may safely be assumed that the variations observed in the vital functions of man in health, contemporaneously with the diurnal revolutions of the earth, play some part, more or less important, in the phenomena of disease. But while many diseases thus manifest certain periodical changes seemingly relative to the diurnal position of the earth to the sun, many also manifest a peculiar periodicity which, so far as time of recurrence is concerned, has no apparent counterpart in the healthy state of the system, and no connexion with the periodical changes going on, either in celestial or telluric phenomena. The periodical variations observed in disease, indeed, are compounded of two very different classes of phenomena, the one of which appertains to changes external to the economy, the other to changes seemingly peculiar to the economy itself.

The writings of Hippocrates are still best referred to in illustration of this last proposition. He asserts that the period of recovery and death in fever is not an indefinite one. Fevers, he states, tend to a "crisis" on certain days. Thus, the mildest class of fevers, and those originating with the most favourable symptoms, cease on the fourth day, or earlier; the most malignant, and those setting in with the most dangerous symptoms, prove fatal within the same period. The second class, as to violence, is protracted to the seventh day, the third to the eleventh, the fourth to the fourteenth, the fifth to the seventeenth, and the sixth to the twentieth. "Thus these periods, from the most acute disease, ascend by fours up to twenty. But [and this observation is of some significance, as we shall see presently] none of these can be truly calculated by whole days, for neither the year nor the months can be numbered by entire days." And then he points out periods, "according to the same progression," of thirty-four, forty, and of sixty days. "In the commencement of these," he says, "it is very difficult to determine those which will come to a crisis after a long interval; for these beginnings are very similar, but one should pay attention from the first day, and observe further at every additional tetrad, and then one cannot miss seeing how the disease will terminate. The constitution of quartans is in the same order."*

Elsewhere he instructs us that "young people for the most part have a crisis in their complaints; some in forty days, some in seven months, some in seven years."†

Further, he fully recognises that many maladies are mainly dependent for their occurrence upon the changes of the seasons, and he inculcates the importance of the physician studying the movements of the heavenly bodies, and the progression of the seasons, so that he may be able to foresee what maladies are most liable to occur, and so be best prepared to fortify the health against them. "Whoever," he writes, "wishes to investigate medicine properly, should proceed thus: in the first place to consider the seasons of the year, and what effect each of them produces, for they are not at all alike, but differ much from themselves in regard to their changes." And again:—"For knowing the changes of the seasons, the risings and settings of the stars, how each of them takes place, he will be able to know beforehand what sort of a year is going to ensue. Having made

* *Prognostics*, § 20. Syd. Soc. Ed. † *Aphorisms*, sec. iii. § 28.

these investigations, and knowing beforehand the seasons, such a one must be acquainted with each particular, and must succeed in the preservation of health, and be by no means unsuccessful in the practice of his art. And if it shall be thought that these things belong rather to meteorology, it will be admitted, on second thoughts, that astronomy contributes not a little, but a very great deal, indeed, to medicine."

It is well to bear in mind that the ancients distinguished the four seasons of the year astronomically, and that Hippocrates is not to be looked upon as wishing—in the fashion of the physicians of a less remote age—to make "the constellations prompt," or "the infant stars confess." Thus, winter began at the setting of the Pleiades, and continued to the vernal equinox; spring at the vernal equinox, and ended at the rising of the Pleiades; summer at the rising of the Pleiades, and ended at the rising of Arcturus; autumn at the rising of Arcturus, and ended at the setting of the Pleiades—the rising and setting of the stars being always understood of what astronomers call the heliacal rising or setting, *i.e.*, when a star rises or sets with the sun.

The doctrine of *critical days* would almost seem to have been placed, by the majority of modern physicians (so little is it now heeded) in the same category as the *quondam* popular, and at one time professional, belief in lucky and unlucky days. The time was when the apothecary stayed for "*choise dayes* or houres," in gathering his herbs and other simples "for the making of drougs and receipts;" and when the physician took heed to certain "perillous dayes" in each month, when prescribing for his patient.

Authorities differ very widely as to the actual days which are perilous. The *Book of Knowledge*, and a monkish rhyme of the time of Henry VI. or Edward IV., quoted by Aubrey,* point out only two days in each month; but a calendar, prefixed to Grafton's Manual or Abridgment of his Chronicle, 1565, is much more liberal. In this instance, the unlucky days are given upon the authority of astronomers, and they are as follows: *January*, 1, 2, 4, 5, 10, 15, 17, 29, very unlucky. *February*, 26, 27, 28, unlucky; 8, 10, 17, very unlucky. *March*, 16, 17, 20, very unlucky. *April*, 7, 8, 10, 20, unlucky; 16, 21, very unlucky. *May*, 3, 6, unlucky; 7, 15, 20, very unlucky. *June*, 10, 22, unlucky; 4, 8, very unlucky. *July*, 15, 21, very unlucky. *August*, 1, 29, 30, unlucky; 19, 20, very unlucky. *September*, 2, 4, 21, 23, unlucky; 6, 7, very unlucky. *October*, 4, 16, 24, unlucky; 6, very unlucky. *November*, 5, 6, 29, 30,

---

* *Miscellanies.*

unlucky; 15, 20, very unlucky. *December*, 15, 22, unlucky; 6, 7, 9, very unlucky.*

The *Book of Knowledge* further tells us that in the change of every moon there are two days in which "whatsoever is begun, late or never, it shall come to no good end, and the dayes be full perilous for many things." From the same source we also learn that astronomers say that six days, to wit, January 3rd, April 30th, July 1st, October 2nd, and December 31st, are perilous of death, and on those days men are forbidden to let blood or take any drink. These six days, we are further instructed, ought to be kept with great diligence, but particularly the 30th of April, 1st of August, and 31st of December, all the veins being at that time full. "For then, whether man or beast be knit in them, within seven days, or certainly within fourteen days, he shall die. And if they take any drinks within fifteene dayes, they shall die; and if they eat any goose in these three dayes, within forty days they shall die; and if any child be born in these three latter dayes, they shall die a wicked death. Astronomers and astrologers say, that in the beginning of March, the seventh night or fourteenth day, let thee bloud of the right arm; and in the beginning of April, the eleventh day, of the left arm; and in the end of May, third or fifth day, on whether arm thou wilt; and thus of all that year, thou shalt orderly be kept from the fever, the falling gout, the sister gout, and losse of thy sight."

But the astrological notion of critical days in health is not to be confounded with the Hippocratic notion of critical days in disease. The former was chiefly a fiction of the imagination, (not entirely, as we shall have occasion to see), the latter was the enduring result of sound observation. "The wit and mind of man," says Bacon, "if it work upon matter, which is the contemplation of the creatures of God, worketh according to the stuff, and is limited thereby; but if it work upon itself, as the spider worketh his web, then it is endless, and brings forth indeed cobwebs of learning [say vain conceits in the present instance], admirable for the fineness [say quaintness, as to the subject in hand] of thread and work, but of no substance of profile."

The doctrine of critical days, uotwithstanding the important truth which underlies it, holds but a very insignificant place in modern systems of physic. This would seem chiefly to arise from an erroneous idea that it is of limited application to the phenomena of disease. One source of misapprehension has probably arisen from the neglect of the fact pointed out by Galen, that crises may *happen on all days*, but that they are

* See Brand's *Popular Antiquities*, Days Lucky, &c.

most common on certain days. Another source of misapprehension may perhaps be traced to a want of sufficient care in observing, or opportunity for watching minutely, the progress of a disease.

"We must confess," writes Van Swietan * (and his observations are confirmed by Cullen), "that physicians do not always use that diligence in the observation of diseases, which we find in the accounts given us by the ancients. They carefully remarked each change which appeared every day of the disease; and by that means found out the manner in which nature cured diseases, following precisely in her footsteps. But who is there that will devote so much of their time to such a tedious observation in each disease? They who have the best opportunities of inquiring into this matter by an ample practice, are often oppressed with such numbers of the sick, that it is impossible for them to attend to every circumstance. There are also a great many physicians who, after noting some observations, and not finding them agree immediately with what has been said on the crisis and critical days, with their indices, by the ancients, altogether neglect them for the future; and believe that, at least in these climates, they are of no use. Though very sensible of my own weakness, and therefore persuaded that my testimony can avail little to so weighty a matter; yet I will venture to declare what in my practice I have observed in diseases, with regard to the present subject.

"In the treatment of acute diseases more especially, not trusting to my memory, I used to write down before the patient everything that I could observe each day through the whole course of the disease; and after returning home, it was my practice to reduce them into order. In this manner have I prepared for myself some hundreds of acute cases through every stage of the disease, remarking at the same time what I had ordered the patient either in way of diet or medicine. I was pleased with this labour, because I could by this means best discover the errors committed in the cure of diseases, and avoid them for the future; and also because I did not dare to call in the advice of our great professor, which I so often found necessary, if I had not an accurate knowledge of disease. For the incomparable Boerhaave, who knew how to frequently exercise a stumbling scholar in more difficult matters, would yet resent with a severe countenance the want of so easy and essential a thing as due attention in a matter which regarded life.

"But when I afterwards compared the maxims of Hippocrates and Galen with what I had thus observed in diseases, I with great pleasure saw the truth of what they pronounced; and I perceived that the chief fault lay in our being so forward to make the ancient physicians wiser and presage more than they intended."

Cullen hit the true key of the subject of critical days when

* *Commentaries*, § 587.

he pointed out that the tendency of continued fevers to terminate at certain periods of their course was probably connected with a general disposition of the animal economy to periodical movements; remarking also that from the universality of *tertian* and *quartan* periods in intermittent fevers, it could not be doubted that there was a proclivity in the economy to observe periods of these types. Many years elapsed, however, before these clear-sighted observations bore fruit.

Galen sought to connect the critical days with the changes of the moon, and Dr. Balfour, in our own day, believed that he had demonstrated the reality of this connexion. It is not necessary that we should do more than allude here to the long debated question of the influence exercised by the moon in determining certain periodical phenomena in man.

It is sufficient also to mention briefly the influence of the seasons upon disease. "All diseases," says Hippocrates, occur at all seasons of the year, but certain of them are most apt to occur and be exacerbated at certain seasons."*

The septenary periods of crisis noted by Hippocrates, were probably derived more from an application of the Pythagorean doctrine of numbers to the phenomena of disease, than from actual observation. To this source is doubtless to be assigned some of the inconsistencies in his mode of stating the doctrine of critical days in different passages of his writings. Not that the doctrine of septenaries altogether lacked confirmation from actual observation, whether applied to the study of disease or health. This was far from being the case. For example, Hippocrates pointed out that such complaints of children as remain, and do not pass away about puberty, or in females about the commencement of menstruation, usually become chronic. He observes also that convulsions do not accompany fever in patients above the age of seven years, and that if they do, they indicate danger. He records, moreover, that several diseases do not attack individuals under the age of puberty, or fourteen years.

The doctrine of septenaries is, indeed, clearly set forth in the Hippocratic writings, the author of which held that the life of man was circumscribed by the number of seven days; and Diocles, the successor of Hippocrates, maintained the same doctrine, and, according to Macrobius, illustrated it in the following manner:—The limbs of the male fœtus are distinct at the seventh week, and the birth takes place at the ninth month, but if they be distinct at the fifth week, birth takes place at the seventh month. If the infant survives the seventh hour, it will probably live; at the end of seven days the umbilical cord sloughs off;

---

* *Aphorisms*, sec. iii. § 19.

in 2 × 7 days the infant perceives the light, and in 7 × 7 days it turns its head to follow with its eyes the objects presented to it. When seven months old, its teeth begin to develop; in 2 × 7 months it can sit without fear of falling; after 3 × 7 months it speaks; in 4 × 7 months it is sufficiently strong to walk firmly; and at 5 × 7 months it has an aversion for the breast. At the age of seven years it loses its first teeth and speaks distinctly; at 2 × 7 years it attains the age of puberty; at 3 × 7 the beard appears, and the youth ceases to grow in height; and at 4 × 7 he ceases to increase in size. In 5 × 7 years the man is at his full strength, and so continues at 6 × 7; but at 7 × 7 the strength somewhat diminishes. Lastly, at 10 × 7 (the two most perfect numbers) are the limits of life; and those who have passed this term are exempt from all labour. We shall presently see that there is somewhat more than a passing and ingenious conceit beneath this doctrine of septenaries.

We have already referred to the association of particular classes of diseases with the different ages of man from infancy to old age, and it remains but to notice those greater cycles of changes, which extend beyond the ages of the individual man, but which are manifested in the multitude, and which are indicated chiefly by outbreaks of pestilence and epidemic diseases. These it has always been sought, from the most ancient periods, to link with the less common celestial and telluric phenomena which recur from time to time, such as eclipses, the appearance of comets and of meteors, and the occurrence of earthquakes, and of extraordinary periods of atmospheric disturbance.

In truth, from a very early age it has been recognised that man was subject to certain periodical changes both in health and disease, a knowledge of which was important as well for the preservation of health as in the treatment of sickness; and amid all the extravagancies of the older physicians we can trace the broad outlines of a grand doctrine of periodicity by which the cyclical changes in man may be harmonized with the cyclical changes observed in celestial and terrestrial phenomena. But notwithstanding the patency of many of the phenomena and their pathological and therapeutical significance; notwithstanding also the prominence accorded to these phenomena in ancient and mediæval systems of physic and philosophy, it is not too much to assert that the question of periodicity, not merely in man, but in the organic world generally, was never submitted to a true scientific examination until very recently.

Apart from the medical aspect of the subject (which, however important practically, is but a fragment of the whole), it must be admitted that the phenomena of periodicity afford the true clue to an accurate knowledge of man's relations to the earth on the one hand, and the celestial bodies on the other—to the COSMOS. To Dr. Laycock belongs the honour of first investigating this subject fully. In 1842, he read a paper before the British Association for the Advancement of Science, entitled, "Evidence and Arguments in Proof of the Existence of a General Law of Periodicity in the Phenomena of Life."* The object of this paper was "to exhibit a general law of vital periodicity, to illustrate that law by pathological phenomena, and to show how it may be investigated and applied to medicine." Dr. Laycock's investigations were prompted first by an inquiry to determine the rule or law by which the interval is regulated between the paroxysms observed in several nervous affections peculiar to women, the symptoms of which recur at regular intervals of time.† In the course of his researches he ascertained that the interval between each menstrual period was not constantly a lunar month, but two, three, four, five, and even seven weeks. He ascertained also that the usual coloured flow alternated every fortnight, sometimes every month, with a white discharge. Facts being such, he determined on counting the intervals by weeks.

The circumstances connected with menstruation led to those connected with the period of utero-gestation in the female. This, it is well known, is usually forty weeks; but here again he found numerous exceptions in the general law. These exceptions, however, allowing for slight inaccuracies in observing, were like those just now mentioned, that is to say, the period was exceeded or shortened by weeks. He then turned his attention to the periods of utero-gestation in lower animals, feeling anxious to learn whether any such limitation of the periods by weeks could be traced as affecting them. He tabulated the periods of gestation in various mammals, and the period of incubation in birds, as they were stated in works on natural history, and as he could make them out from personal inquiries whenever opportunity offered. Rigid accuracy could not, of course, be looked for in observations of this kind, but such as they were, they confirmed the general law of limitation by weeks observed in the sex. He collected tolerably trustworthy observations of this kind, referring to one hundred and twenty-nine species of birds and mammals (some being, indeed, rigidly exact), and in sixty-seven of these

---

* This paper (with additional observations and inquiries) will be found in vol. i. of the *Lancet*, 1842-43 (see pp. 124, 160, 423, 929); and in the second volume of the same journal for that year (see papers on *Lunar Influence*, pp. 438, 826), as well as the first volume for the year 1843-44 (see papers on *Annual Vital Periods*, p. 85, *Major Periods of Development*, p. 253, and *On Periods of Years*, p. 430), the subject is still further examined.

† See his *Treatise on the Nervous Diseases of Women*. London, 1840.

the periods of utero-gestation and incubation were a definite number of weeks or months: twenty-four exhibited periods being within a day of a definite number, and in the remaining thirty-nine, the period was so loosely stated as not to be of much weight either for or against the general law, although by far the greater number were decidedly favourable. Altogether he judged there were only four available exceptions. As examples of this law, it may be stated that in the grallidæ, tetraonidæ, and other birds about the same size, the period of incubation is three weeks; in the anatidæ, four weeks; the cygnidæ, six weeks; but in small birds, as the musciparæ, only two weeks.

Dr. Laycock now extended his inquiries into the habits and physiology of animals lower in the scale. He found only two instances in which the period of incubation in fishes and reptiles was stated. Dr. Knox ascertained that the period of incubation or hatching of the ova of the salmon was exactly one hundred and forty days, or twenty complete weeks. The other example is the aquatic salamander, the ova of which are hatched in fifteen days.

But the most remarkable illustrations and confirmation of the law were found in insects. The changes to be noted as being regulated, as regards the time they occupy by this law, were the following: 1. The hatching of the ova; 2. The caterpillar, or larva state, and the moults which take place at that stage of development; 3. The pupa, or chrysalis period; 4. The imago state, or puberty.

The ova are hatched in periods varying considerably in length. The shortest is half a week, or seven lunar days, as in the wasp, the common bee, and ichneumon; in some, as *cecidomia tritici*, the period is one week; in others, it is a week and a half, as for example, the black caterpillar and the gooseberry grub (*tenthredo capræa*). In the majority of insects, however, it is from two weeks to six weeks. The ova of the glow-worm occupy six weeks, of the mole-cricket only four weeks, in hatching. The period passed by insects in the larva will vary in length as the insect varies; but Dr. Laycock thinks it is seldom less than seven days. In the common bee it is six days and a half, in the humble bee seven days exactly. In day-papiliones it is four weeks, in moths six weeks. In many insects it is a long period, continuing for months. The larva of a new British wasp, of the genus oplopus, occupies twelve weeks, namely, from the period when its first two segments coalesced to the throwing off of its exuviæ was three weeks, and from the time of the latter change to its full development, nine weeks. It is worthy of notice that the time occupied between each exuviation of the larva is limited in the same manner as the period of the larva state itself is limited. Thus, the latter period of the common black caterpillar is twenty-one days, or three weeks: during this period it exuviates or changes its skin three times, at intervals of seven days each. The wood-piercer bee is in a larva state four weeks; of these four weeks it fasts exactly one, just before it enters the pupa state.

The period spent in the pupa state is most in accordance with the general law of limitation by weeks; and Dr. Laycock observes that the more exact the observations are as to the length of this period, the more confirmatory are they of the general rule. For example, Mr. Denny had three larvæ of the *sphinx atropos,* which went into the earth on August 22nd, 24th, and September 2nd, respectively. They appeared as perfect moths on October 16th, 18th, and 27th, or in each case in exactly eight weeks.

Few observations have been made as to the duration of the imago state, but such as exist, indicate that the vital actions of the perfect insect appear subject to the same general law.

There are various changes and functions in animals also amenable to it—for example, the moults of insects, as of the *arachnida, myriapoda,* and *crustacea,* the exuviation of serpents, and the renewal of the dermoid appendages in birds and mammals. The minor processes in the last mentioned are, moreover, regulated by the same law. Thus the ring-pigeon not only sits fourteen days, but lays eggs, previous to sitting, for fourteen days. The female of the great seal calves on the rocks, and suckles its young at every tide for fourteen or fifteen days, when the calf casts its coat, and goes into the water.

These facts, Dr. Laycock observes, are general facts, and cannot happen day after day in so many millions of animals, of every kind, from the larva or ovum of a minute insect up to man, at definite periods, from a mere chance or coincidence; and although temperature, food, domestication, and other modifying circumstances, may and do interrupt the regularity with which the various processes he has alluded to are conducted, yet upon the whole it is, he thinks, "impossible to come to any less general conclusion than this, that in animals, changes occur every three and a half, seven, fourteen, twenty-one, or twenty-eight days, or at some definite number of weeks."

Having made good his ground by collecting the facts in physiology and natural history bearing upon the periodicity of vital movements, Dr. Laycock entered upon a consideration of this periodicity as exhibited in disease, confining himself principally to the large class of febrile diseases, and certain points in the pathology of gout.

And first of *small-pox* (to begin with the *exanthemata*). In distinct small-pox, when it pursues its regular course, there is (1)

a febrile stage, from the first to the fourth day, (2) an eruptive stage, from the fourth day to the seventh, (3) the ripening, from the seventh to the eleventh; and (4) the stage of desiccation, from the eleventh to the fourteenth or fifteenth (Dr. Craigie). Anomalous small-pox, vaccinia, and varicella, follow the same order of development, as well as rubeola and scarlatina. Thus the rash of measles appears on the fourth day, and declines on the eighth. Exanthematous typhus appears to be as regular in its stages as variola, but differs in exhibiting the tertian type rather than the quartan. Other examples are cited by Dr. Laycock.

Intermittent fevers display the influence of a law of vital periodicity, more distinctly, perhaps, than any other class of febrile disorders. There are three principal forms, the quotidian ague, the paroxysm recurring every day; the tertian, every third, counting the day on which the preceding paroxysm occurred; and the quartan, in which there are two entire days of freedom from fever between each paroxysm. The tertian, according to most writers, runs itself out in seven paroxysms, occupying fourteen days, the *interval* between each paroxysm being forty-eight hours. The hemi-tertian, double tertian, alternate tertian, and quartan, &c., are compounded of these three primary forms.

Now, as we expect similar effects from similar causes, Dr. Laycock remarks, it is no irrational inference that since intermittents present so constant and regular an order in their progress, their cogeners, the remittent and malignant fevers, will exhibit, *mutatis mutandis*, a similar regularity. The regularity thus theoretically inferred has been actually observed for many centuries, and has given rise to *the doctrine of critical days in fevers.*

"It is very obvious," writes Dr. Laycock, "that the writer who first laid down this doctrine *in extenso*, attached a meaning to the term differing very considerably from the modern meaning, or even from the notions of Galen. In the essay on the judicatory or critical days found among the writings of Hippocrates, a critical day is shown to be that day on which certain symptoms will appear, enabling us to ascertain —first, the probable duration or termination of the disease; and secondly, the symptoms likely to appear on certain future days. The act of mind which deduced these inferences was termed a judication (judicationes— *κρίσιες*), and the days on which that act was to be made were termed judicatory (*κρίσιμος*). So a day might be judicatory, first of the disease, its course and termination; secondly, of the symptoms to happen on another day. Thus jaundice and hiccups appearing on the fifth day of fever, indicated a fatal disease; jaundice, on or after the seventh, indicated diaphoresis; on the seventh, ninth, eleventh, and fourteenth (if unaccompanied by hardness in the præcordia), a favourable termination. In pleurisy, if the fever abates on the seventh day, the patient will recover; if it do not, the disease will be prolonged to the eleventh or fourteenth, on which days it is often fatal. This is the first and plainest exposition of the doctrine of critical days, and, I believe, is perfectly correct. If Hippocrates had described the critical days in small-pox, it would have probably been after this manner:—'in a fever commencing with rigors, pain in the præcordia and vomiting, if pimples appear on the fourth day, they will begin to suppurate after the seventh; on the eleventh the swelling and inflammation will manifestly abate, and quite disappear on the fourteenth.' If the swelling and inflammation do not abate on the eleventh day, death will take place on the fourteenth. This is something like what a Hippocratic aphorism would have been respecting small-pox. Similar examples might be drawn from the prognosis of tertian fevers. . . . . ."

Having thus elucidated the doctrine of critical days, and placed it in the simplest point of view, Dr. Laycock next endeavours to make good his comparison of the critical days of febrile diseases, and the order of sequence observed by intermittents; and further, he compares both these forms of fever with the periodicity observed in the exanthemata, and seeks to make the facts bearing upon that part of their pathology harmonize with each other:—

| | | | | | | | | | | | |
|---|---|---|---|---|---|---|---|---|---|---|---|
| "The critical days, according to Hippocrates, are— | 1 | 4 | 7 | 9 | 11 | 14 | 17 | 20, or 21. | | | |
| The paroxysms of a tertian will take place on the— | 1<br>1p. | 3<br>2p. | 5<br>3p. | 7<br>4p. | 9<br>5p. | 11<br>6p. | 13<br>7p. | 15<br>8p. | 17<br>9p. | 19<br>10p. | 21<br>11p. |
| The paroxysms of a quartan— | 1<br>1p. | 4<br>2p. | 7<br>3p. | 10<br>4p. | 13<br>5p. | 16<br>6p. | 19<br>7p." | | | | |

"And if a continued fever (Dr. Laycock adds) existed with tertian or quartan exacerbations, the more violent symptoms might be expected to appear on the days indicated. On comparing the order of days, discrepancies between the three are sufficiently obvious on a superficial consideration, but many of them disappear on more particular inquiry. Supposing a remittent fever has the quartan type, and that after the fourth paroxysm an intermission takes place, on what day would the amendment appear? Clearly on the eleventh; and the change of symptoms on that day would be judicatory of a mild paroxysm on the thirteenth, and final amendment on the fourteenth. If the intermission occurred after the fifth paroxysm, then the fourteenth would be the critical day; if at the sixth, the seventeenth; if at the seventh paroxysm, the fever ends on the twentieth day. Some fevers, however, have the tertian, others the quotidian type; and this leads to the observation that there are certain days to be distinguished from the rest. Whatever type the fever may exhibit, there will be a paroxysm on the seventh day, and consequently this day should be distinguished by an unusual fatality or number of crises. For analogous reasons the fourteenth will be remarkable as a day of amendment, the last paroxysm of a quotidian taking place on that day, and the last of a tertian on the day previous; for observation has established that if a tertian is to cease about the fourth paroxysm (the seventh critical day), the second paroxysm will be more severe than the first or third; but if the fourth be severe, and the fifth less so, the disease will end at the seventh paroxysm, and, of course, the change for the better, if the rule be applied to remittent or continued fevers, will be seen on the fourteenth day. Should, however, the exacerbation occurring on the thirteenth day end fatally, whether it be the seventh of a tertian or the fifth of a quartan, death will probably take place early on the fourteenth day, namely, about three or four o'clock a.m., when the system is most languid."

If it be asked, are these theoretical inferences respecting the seventh and fourteenth day, corroborated by what actually occurs in fevers, Dr. Laycock answers in the affirmative, and his opinion coincides with that of all writers who have written on the subject.

As to the exanthematous fevers, it will be seen at a glance that the "critical days" they exhibit occur in quartan order. Exanthematous typhus exhibits the tertian type, and the critical days in this fever are the fifth, seventh, ninth, eleventh, and twenty-first. Scarlet fever is sometimes tertian, sometimes quartan.

Dr. Laycock further illustrates the law of vital periodicity from the pathology of a paroxysm of gout, and he expresses the opinion that the doctrine of critical days can be traced in acute, and even chronic diseases, (as its first propounders held,) when dates are accurately given.

Now, it will have been noted that *a period of seven days,* and *definite fractions or multiples of that period,* are very prominent in the series of phenomena Dr. Laycock has directed attention to, and as he remarks,—"It is a direct inference that the doctrine of 'septenaries,' so much considered in ancient philosophy, [and to which we have already referred], was based upon similar observations." Moreover, he holds that the use of the number *seven* among ancient physicians "was closely connected with the principles of a science, or more properly a *system of vital proleptics,** now utterly lost, and which was founded on the observation of periodic, vital, and meteorological phenomena. The remains of this science," he adds, "appear (as if fossilized) in the quackery of nativity casters, astrologers, &c."

Dr. Laycock next endeavours to harmonize the physiological and pathological facts which he has adduced, and first he points out that it is obvious there are "critical days" in health,—days in which there is a marked change in the vital movements, whether that change be for the better or worse, and that those days may

---

* *Proleptics, Proleptice,* from πρὸ, "before," and λαμβάνω, "I seize hold of"—the art and science of predicting in medicine. The term was first proposed by Dr. Laycock.

be stated generally as the fourth, seventh, eleventh, fourteenth, twentieth, (or twenty-first,) and twenty-eighth. If, he says, we examine into the nature of the changes which occur at these periods in various animals, we find that they consist essentially in a movement inducing a less healthy state of the system at large. The period of moulting of all animals, whether insects, birds, or mammals, is a sickly period ; a period in which fatal disease often commences. It is well known that the menstrual period is of this character; and, although there is no manifest change of this kind to be observed in men generally, yet it has been often observed that the male sex are subject to periodic changes in the tone of the system, during which there is a greater liability to disease, greater irritability of the system, and when, in fact, disease (in those predisposed) exhibits itself exclusively. Of this kind are numerous nervous affections; some so slight as scarcely to be observable, but some of a graver character, and therefore marking the periods distinctly. In these instances the periods harmonize with the law of limitation by weeks. A like conclusion is derived from an examination of the latent period (the period of incubation) of fevers. Depression of the system, in fact, takes place at the critical days of health, and consequently an exanthem, or other fever depending on a poison, is more likely to appear on those days than any other ; and by parity of reasoning the symptoms are most likely to be violent (when the disease has once broken out), or, in other words, a paroxysm to appear, on the same days ; consequently, the critical days of health and the critical days of fever will be identical.

"And as a confirmation of these views," Dr. Laycock adds, "it may be observed (as *a priori* might be inferred) that the latent period of fevers rarely extends beyond twenty-eight days. If we take menstruation as a type of the critical days, and suppose that a movement takes place every seven days, gradually becoming more intense at each up to the fourth week, we have four days, at least, in every month in which the peculiar symptoms of the poisons, whether malarious, exanthematous, or contagious, may exhibit themselves ; probably the number is greater; but if one or two of the days be passed over without an outburst of febrile action, it is scarcely possible that the third or fourth will. And in strict accordance with these theoretical inferences we find most writers stating the latent period of febrific poisons."

We reluctantly pass over the further application of this doctrine to the observation of febrile disorders ; but the following brief remarks on its bearing upon the treatment of disease may be quoted with advantage;—

"There has been much difference of opinion as to the most efficient mode of treating fevers, which a consideration of this law may elucidate.

From the preceding statements it may be readily inferred that fevers, like gout, will get well of themselves. If a physician were to give simple remedies to his fever patients on the sixth or thirteenth day, he would probably find the amendment as astonishing on the seventh or fourteenth, as if he had administered the most vaunted febrifuges. Nor does this observation apply to febrile affections only ; it holds good with the greater number of idiopathic diseases, as they are termed. One might almost venture to assert that the *scientific* observation and treatment of disease are impossible, without a knowledge of the mysterious revolutions continually taking place in the system."

The facts drawn from natural history, upon which Dr. Laycock's induction is founded, that there is a law of the kind he has discussed, governing all the vital changes of all animals, are, as he rightly observes, all the more satisfactory, because collected from a variety of sources, and ascertained by a multitude of observers, to whom the existence of such a law seems to have been unknown.

His further observations refer to quotidian and tertian intervals, as well as to the hour of the day at which they occur, the greater periods observed in vital phenomena (lunar, seasonal, annual, &c.), and to the relation of both the major and minor periods to external phenomena. He states that the periodicity observed in man may be connected, either with cyclical processes inherent in the system (*esoteric*), or dependent upon periodic agencies acting from without (*exoteric*), or result from a combination of the two (*endexoteric*). We do not propose to follow Dr. Laycock in his examination of the relations which exoteric may bear to esoteric agencies in the phenomena of periodicity, neither shall we dwell upon his investigation of the lunar, seasonal, and annual cycles, and only so far upon the diurnal, as to make clear the meaning of one of the conclusions with which we shall terminate this necessarily imperfect reference to his writings on the subject of this article. The complete day of twenty-four hours is the pathological period most generally noticed by physicians, but Dr. Laycock shows that there are periods of three days and a half, or seven half days. He thinks, therefore, that the half day period should be adopted as the period which will best comprise the phenomena of the most marked class of periodic diseases, the intermittents. He reverts, indeed, to the ancient division of the whole day, or *nyctemeron*, into two parts. Dr. Graves is, perhaps, the only physician who has made the same observation, and applied it to pathology. He observes that if this period were made use of, " we should not count three days and a half, but seven half days ; we would not say seven days, but fourteen half days. If this method were adopted, many of the apparently anomalous critical effects and critical terminations in continued fevers would, I have no doubt, be found strictly conformable to some regular law of periodicity."

Dr. Laycock sums up his conclusions on a General Law of Vital Periodicity thus:—

"1. That there is a general law of periodicity which regulates *all* the vital movements in *all* animals.

"2. That the periods within which these movements take place admit of calculations approximatively exact.

"3. That the fundamental unit—the unit upon which these calculations should be based—must for the present be considered as one day of twelve hours.

"4. That the lesser periods are simple and compound multiples of the unit in a numerical ratio analogous to that observed in chemical compounds.

"5. That the fundamental unit of the greater periods is one week of seven days, each day being twelve hours; and that simple and compound multiples of this unit determine the length of these periods by the same ratio as multiples of the unit of twelve hours determine the lesser periods."

Dr. Laycock's papers on periodicity were, he tells us, presented to the world rather as "*mémoires pour servir,*" than as finished productions. They were mainly intended to form the outline of a science of *Vital Proleptics* (having for its object to *foretell social and individual suffering*), and "to incite the collection of materials for the complete structure scattered through various branches of science, and particularly to point out what observations are wanting to connect and cement these materials." It is much to be regretted that these papers were never published in a separate form; and it is perhaps not too much to say that the instructions which Dr. Laycock gives for the observation of periodical changes in disease, in his work on *Medical Observation and Research,* and the philosophical consideration of periodicity in vital phenomena in his more recent work on *Mind and Brain,* fail of their legitimate effect upon the student and reader from lack of a preliminary acquaintance, or of ready access, to the detailed researches in the papers which we have briefly glanced at. The new impetus which will be given to the study of periodicity in health and disease, by the publication, in a compendious form, of Dr. Ed. Smith's researches, will, we hope, induce Dr. Laycock to collect his papers on vital proleptics, and publish them as a separate work.

We have confined ourselves to those portions of Dr. Laycock's researches which led to the enunciation of a general law of periodicity, derived from the observation of many physiological and pathological phenomena in the animal kingdom, and in man.

Our purpose has not, however, been merely to direct attention to the law deduced by Dr. Laycock, but also, and in an especial manner, to the method by which he arrived at that law. This is full of the most valuable instruction for the further study of the phenomena of periodicity.

We do not follow Dr. Laycock in his examination of the diurnal, seasonal, and annual periods observed in man, as indicated by certain physiological and pathological phenomena recorded by many observers, since the physiological observations, confessedly imperfect, have been superseded by the important researches of Dr. Ed. Smith, the results of which are embodied in the volume referred to at the commencement of this article. These researches differ from, and surpass, all previous experimental observations on the subject, in comprehensiveness, completeness, and extent; and they include the *daily*, *weekly*, and *seasonal cycles*, together with an important series of observations on the *cyclical changes in the Ages of Man*, and a most suggestive examination of the question concerning the *cycle of the Generations of Man*.

In the section on the *Daily Cycle*, after referring to the researches of M. Quetelet, Dr. Knox, Dr. Guy, and others, on the diurnal variations in the rate of pulsation and respiration, Dr. Smith justly says that their inquiries

"—Were deficient in that they did not in any case embrace the whole cycle of the twenty-four hours, and were not made under conditions so precisely parallel that they could be advantageously compared with each other, and hence it was desirable to make such a new series of observations as by their duration, the number of persons under observation, the uniformity in the conditions of the inquiry, and the extreme regularity in the period and mode of making the observations, should afford complete cycles and conditions which should render the results entitled to credit. To this end two sets of inquiries were set on foot, one in reference to health, and the other in cases of phthisis; the former embracing sundry observations on five persons, including children, and extending over three days and nights, and the latter on six adults of both sexes, extending over six days and nights without intermission. The general conditions imposed were, precision in the hours of meals, and of rising and retiring to rest, absolute rest during at least five minutes before each inquiry, and rigid attention to the hour, to the order of the cases, and to the method of counting and registering the rate of functions. . ." (p. 5.)

Under these conditions Dr. Smith sought to determine the rate of pulsation and respiration, the amount of carbonic acid expired, and air inspired, also the quantity of urea and urinary water evolved, and with the following general results :—

The rate of both pulsation and respiration was increased during the day, and decreased during the night; and as these changes did not occur abruptly, there was a period of increase in the morning, and of decrease during the evening. There were great and rapid variations of the rate at the several hours of the day, and these were much greater in the day than in the night, there being three or four marked elevations in the former period, alternating with an equal number of depressions. "The elevations follow the various meals, and are commonly the highest after the breakfast and tea, and within two or three hours after the meal. The depressions precede the taking of food, and of these, that which precedes the breakfast is by far the greatest. The periods of lowest pulsation during the day were commonly 8 a.m. and midday, or the periods immediately preceding the breakfast and dinner." (p. 9.) An increase was also observed during the act of eating, which disappeared in the intervals of the courses.

The general course in the daily cycle of pulsation and respiration was as follows:—

"In the evening from 7 to 9 p.m., there is an evident tendency in the rate to decline, and with some slight variations this is continued progressively through the following hours until from 1 to 3 a.m., when the rate is at its minimum. During the next two hours there is a slight tendency to increase, but it is very gradual until the usual hour for rising, when it will have attained an increase of several pulsations per minute. Immediately after the breakfast has been taken there is a rapid and great increase, which attains its maximum in the second hour afterwards, after which it declines greatly in an hour, and loses from ten to fifteen pulsations immediately before the dinner. After the dinner has been taken there is another increase, but the rate is seldom raised so high as that which follows the breakfast, and the highest point is attained in the second or third hour. This again is followed by a decrease which precedes and a subsequent increase which follows the tea, when a point as high as that which follows the breakfast is usually found [our readers will remember Sir Gawain]; and lastly there is the final decrease which is usually progressive notwithstanding that supper may be taken at a later hour. When dinner had been taken at a later hour than that above indicated, the rate of the functions followed the same course as that now given, except that there was not any important increase after midday until the dinner-hour. The rate remained low, but not uniform, from 12 to 1 p.m. until the dinner-hour." (p. 11.)

The experiments to determine the amount of carbonic acid expired, were of rare interest in their conception and execution, and the results showed a striking similarity between the amount evolved and the variations in the rate of pulsation and respiration—"so striking, indeed, that the curves might almost be taken to represent both the rate and the quantity." There were commonly four minima and three maxima in the daily quantities of carbonic acid evolved, the former found immediately before each meal (except supper) and during the night, and the latter following each meal. The largest increase commonly followed breakfast and tea, and then the total quantities evolved were nearly identical, whilst there was also a great similarity in the minimum quantities recorded at the end of the interval between the meals. This variation was due to food, but there was a low point below which the quantity did not fall.

There was a general concurrence in the progression of the quantities both of the carbonic acid expired and the air inspired. Thus, in these various series of inquiries, "results of very close uniformity were obtained, all agreeing in the marked difference in the vital actions in the day and the night, the great and rapid influence of food, the temporary effect of food, and the varying amount of action due to different meals." (p. 34.)

The conclusions respecting the evolution of urea were deduced from an elaborate series of observations extending over several months, and showed that the greatest amount of urea evolved per hour is in the early part of the day. As the day advances, the amount declines, whilst the lowest amount of the twenty-four hours occurs during the night hours. The maximum period of the day is about, or even after, midday; and the ascent and descent in the quantity are very rapid. A second increase was observed at 6 or 8 p.m. The circumstances under which the experiments were performed as to food showed, "that the larger evolution of urea neither corresponded with the amount of nitrogenous matter taken in the previous meal, nor with the exertion made, but rather with the degree of activity of the vital processes." (p. 39.) The quantity of urinary water eliminated by the kidneys corresponded somewhat with the quantity of urea evolved per hour.

Under the influence of *fasting* it was noted that whilst the daily cycle of changes in the carbonic acid expired, and air inspired, were reduced almost to a nullity, there was a progressive decrease in the rate of pulsation and respiration, and in the quantity of vapour evolved by the lungs, "but in all these points of inquiry alike there is a lower condition existing in the night than the day." The hourly excretion of urine followed precisely the same course as when food had been taken.

These results incontestably make manifest that there is more vital action going on at one period of the day than another, and that the morning is the period of greatest vital action.

An extension of the experiments over a series of weeks proved that the intervention of a day of rest on the Sunday, gave rise to a distinct septennial period, characterized by indications of invigorated powers on Monday (very significant of the physical importance of a day of rest), and showing at least that an influence acting periodically, was accompanied by periodical effects.

The effects of the *Seasons* upon the evolution of carbonic acid, the quantity of air inspired, the depth of inspiration, and the rate of pulsation and respiration, the elimination of urea, water, and other excretions, were determined by Dr. Smith in a remarkable series of experiments, extending from the early part of 1858 to the same period in 1861. We shall state the summary of results in his own words; but this, as often happens, conveys but an imperfect notion of the true value of the results.

"*Summer*.—The summer season exerts the most marked power, and under its influence the body exhibits the following minimum and maximum conditions. There is the *minimum* of carbonic acid and vapour exhaled, of air inspired, of the rate and force of respiration, of alimentation and assimilation, of animal heat generated, of muscular tone and endurance of fatigue, and in general of resistance to adverse influence. There is the *maximum* of the rate of pulsation, of the action of the skin, and the elimination of vapour, of the dispersion of heat, of the supply of heat from without, and of excess of heat, of elimination of urea and urinary water, of the distribution of blood to the surface, of the imbibition of fluids, of relaxation of the tissues, and of poverty and carbonization of the blood.

"*Winter*.—In the winter season the above conditions are, for the most part, reversed.

"*Autumn*.—Autumn is marked by the summer or the winter condition, as the character of the season resembles the one or the other; but it is essentially a period of change from the minimum towards the maximum of vital conditions.

"*Spring*.—In the early and middle parts of the spring season every function of the body is in its highest degree of efficiency, but as it advances, these maximum conditions emerge into those of summer.

"Hence the effect of season *is more than the physical phenomena of temperature and atmospheric pressure explain*,* and is so universal that even the same amount of exertion made at two different seasons produced different degrees of effect upon the vital changes—less carbonic acid being evolved from it in summer than in winter in proportion to the relative amounts when at rest at those two periods." (p. 158.)

We pass over Dr. Smith's observations on the *Cycle of the Generations of Man*, although by no means the least important or interesting portion of his work. In fact, whether looked upon in a scientific or practical point of view, the treatment of this portion of his subject would alone suffice to establish the unusual value of Dr. Smith's work.

On the *Cycle of the Generations of Man* but few observations

* The italics are our own.

are here needed. Under this head Dr. Smith considers the occurrence of epidemics as evidence of rapidly recurring periods of unusual liability to disease; and variations in the habits, wants, and dangers of society, particularly as bearing upon changes in the condition of the human constitution as indicated by change in the type of disease. The essential idea in an epidemic outbreak is, as Dr. Smith points out, "that of a disease which attacks mankind with *unusual* prevalence, or with *unusual* virulence, so that a large mass of the people become afflicted by it, or large numbers die from it." Now, the unusual prevalence and the great mortality, he adds, "clearly indicate the occurrence of causes of disease acting temporarily with unusual power, or a state of system which is unusually deficient in its power to resist the causes of disease, or it may be that both these conditions occur at the same time." In illustration of these points he gives a brief survey of the great epidemics of the Middle Ages.

The great value of Dr. Smith's researches, so far as our present subject is concerned, consists in the scientific determination of the nature and character of the daily and seasonal cyclical changes observed in man. The *fact* of these changes has been known from the earliest ages, their nature had been surmised by previous observers, but the honour of determining their true character must rest with Dr. Smith.

It is difficult to exaggerate the importance of these researches, both in their scientific and their practical results. In the former respect, they throw a flood of light upon the diurnal and seasonal phenomena of disease; in the latter, they furnish rules of incalculable importance for our guidance in the preservation of health and the treatment of diseases. These points are treated with admirable perspicacity by Dr. Smith, and his work, as a consequence, should become simply a necessity to the scientific physician. On this account we shall only briefly glance at the practical and bulkiest portion of the book. He discusses the influence which a knowledge of the daily cyclical changes should exercise upon the times of taking food, upon the nature of our clothing, upon the periods of mental and physical labour, and the use of remedies. He shows in what manner this knowledge bears upon our insight into the periods of invasion and exacerbation of diseases, and enables us better to appreciate the importance of a study of those diurnal phenomena of disease which we have already set forth from the writings of Virey. He shows, also, that it is of importance not to neglect a consideration of the period of the day in our administration of the different classes of remedies, and lays down rules for our guidance in this respect.

In like manner, after describing in detail the influence of the seasonal cycle on muscular power, sensibility, the viability of children born in different months, and growth, he discusses its effects upon alimentation, the liability to and the cure of disease, and the rules of conduct to be derived from a consideration of these points. His observations enable us for the first time clearly to comprehend the sources of the seasonal variations in the prevalence of different diseases, and the importance of a regard to seasonal peculiarities in the treatment of disease. "The rotation of the seasons," he observes, "is a chief element of the *vis medicatrix naturæ;*" and he illustrates this proposition with great force from the periods of prevalence and cessation of plague and cholera. "The precise progress of the epidemic," he shows, "is that of the varying powers of the system. It begins as they decline, increases as they decrease, is the greatest when they are the least, decreases as they increase, and disappears when they have recovered the height from which they fell." (p. 216.) Not the least important result of Dr. Smith's observations consists in giving us a trustworthy clue by which to investigate the influence of a cycle of years upon the causation of epidemics.

It is no part of our purpose to seek to harmonize the results of experimental research and pathological observation on the phenomena of periodicity in man. The results of both series of investigations approximate at several points, particularly in the diurnal and seasonal cycles ; and they have this in common, that they alike indicate periodical oscillations in the degree of action of the vital powers. For the doctrine of *critical days in health,* as taught by Dr. Laycock, is as legitimate a deduction from pathological and physiological phenomena, as the nature and progress of the cyclical changes observed in the diurnal and seasonal periods, described by Dr. Smith, are sound conclusions from experiment. We have evidently much to learn of the cyclical processes inherent in the system, as manifested in certain pathological states ; and it becomes an interesting question whether experimental research will aid us still further in solving the difficulties in our way, and particularly in throwing additional light upon the doctrine of critical days. But be this as it may, it is not to be forgotten that the deductions from experimental research do not set aside those derived from pathological observation. Our object has been, however, to draw increased attention to the importance of studying the phenomena of vital periodicity, and if the method we have adopted is somewhat disconnected and fragmentary, we trust, nevertheless, that it will not be the less calculated to further the end we had in view.

The publication of Dr. Smith's book will not only constitute an important epoch in our knowledge of vital periodicity, but it fully justifies us in asserting (what Dr. Laycock nearly twenty years ago almost ventured to assert), that "the *scientific* observation and treatment of disease are impossible without a knowledge of the mysterious revolutions continually taking place in the system."

---

### Art. IV.—ON HALLUCINATIONS IN THEIR RELATION TO MEDICAL JURISPRUDENCE.

